# Experiences of simulated patients in providing feedback in communication skills teaching for undergraduate medical students

**DOI:** 10.1186/s12909-022-03415-6

**Published:** 2022-05-03

**Authors:** Riya Elizabeth George, Harvey Wells, Annie Cushing

**Affiliations:** Barts and The London School of Medicine and Dentistry, Queen Mary University of London, Robin Brook Centre, St Bartholomew’s Hospital, London, EC1A 7BE UK

**Keywords:** Simulated patients, Feedback, Clinical communication, Undergraduate medical education

## Abstract

**Background:**

Simulated Patients (SPs) are commonplace in the teaching of communication skills in medical education and can provide immediate feedback to students from the patient’s perspective. The experiences of SPs and their perspective on providing feedback is an under-studied area. This study aims to explore SP experiences and views on feedback, factors influencing their feedback and implications for training.

**Methods:**

Using a constructivist grounded theory approach, we conducted six focus groups with 30 SPs. Participants included experienced simulated patients from a London-based actor agency, used in undergraduate teaching programmes of communication skills. Consistent with the principles of grounded theory, data was collected and analysed in an iterative process to identify themes.

**Results:**

Five over-arching themes were identified: 1.) Feedback processes, 2.) Challenges in providing feedback, 3.) Cumulative experiences, 4.) Web of interpersonal relationships and dynamics and 5.) Portraying the character and patient representations.

**Discussion:**

These SPs regarded the sharing of the emotions they experienced during the consultation as the focus of their feedback. Their preference was for giving a ‘sandwich style’ of feedback and ‘out-of-role’ approach. The relationship with facilitators and students and politeness conventions emerged as significant factors when providing feedback. Sensitivity to the social dynamics of groups and implicit facilitator expectations were challenges they experienced as was divergence in views of student performance.

**Conclusion:**

This study explored SP experiences and perspectives on providing feedback. Findings reveal complex social and structural dynamics at play in providing feedback which have not been reported so far in the literature. It is recommended that these issues should be addressed in training of both SPs and facilitators, in addition to feedback guidelines.

**Supplementary Information:**

The online version contains supplementary material available at 10.1186/s12909-022-03415-6.

## Background

Simulation with human role players, interacting with learners in a wide range of experiential learning and assessment contexts for developing effective communication skills, is a recognized methodology in medical education [1–5]., A SP is a person who is trained to accurately portray the characteristics of a specific patient in a realistic way, which is sometimes standardised to deliver a consistent presentation that does not vary between students [5]. Feedback from the patient’s perspective provides students with unique and valuable information about how the student’s behaviour impacts SPs emotional experience of the encounter, trust in the student and understanding of information [6]. These simulations are commonly observed by the students’ peers and a facilitator, allowing for discussion and feedback for learning.

Medical students highly value the feedback provided by SPs [7, 8] and training with SPs can enhance students’ communication [9–12]. Whilst the value of their feedback is generally recognised, knowledge about the most effective ways in which SPs can provide feedback is scarce [13, 14] and wide variation in SP training across medical schools has been reported [5]. A recent consensus of standards of best practice for standardized/simulated patient educators has been published by a panel of SP expert educators, known as the Association of Standardised Patient Educators (ASPE) in the field of SP methodology [15]. This document outlines precise guidelines whilst recognising the need for flexibility to address the diversity of varying contexts of SP practice. Table 1 outlines the key principles relating to ‘feedback delivery’. SP educators should follow in relation to SP training methodology. Despite, the fact that simulation programmes have been running for decades there is a lack of research into SP experiences and views on their feedback practice.

**Table 1:** The Association of Standardized Patient Educators (ASPE) Standards of Best Practice (SOBP)

Principle	Practice
3.3 Training for feedback	3.3.1 Review with SPs the fundamental principles of feedback as they relate to the planned activity. 3.3.2 Inform SPs of the feedback objectives and level of the learners with whom they will be learning. 3.3.3 Inform SPs of the feedback logistics and setting (e.g., one-on-one feedback with learner, small group feedback, simulation debrief). 3.3.4 Train SPs to use their observations, responses, and knowledge to provide feedback on observable, modifiable behaviours in learners. 3.3.5 Ensure SP readiness through repeated practice and targeted feedback.

### Providing feedback from a patient’s perspective

Feedback in medical education is defined as ‘specific information about the difference between a trainee’s observed performance and a given standard, with the target of achieving improvement in the performance of the trainee’ [14]. Recent definitions of feedback recognise it as a dynamic process with emphasis on the learner’s self-assessment and where feedback is conceptualised as a social negotiation enacted in the context of a relationship [15–17]. Medical educators have begun to recognise contextual and socio-cultural factors such as the ‘educational alliance’ (quality of the relationship) between teachers and learners, perceptions of credibility, learner receptiveness to feedback and the institutional learning culture towards feedback [18–20]. However, within this contextual shift, research exploring the experiences of SPs and their essential role in providing feedback is lacking. Existing literature suggests SP feedback is highly variable in terms of its content, the language used and the quality [13]. The variables influencing SP feedback are relatively unknown. As SPs’ contribution in medical teaching and assessment increases, further research is warranted to explore their experiences and perceptions of how the ‘patient perspective’ can be effectively conveyed in their feedback.

### Research aims

The research questions explored in this study were:

1. What are the experiences and perspectives of SPs regarding their role in providing feedback?
2. How do SPs construct their role based on their feedback experiences?
3. How are SPs situated in the learning environment?
4. What implications arise from SP’s experiences and perspectives on providing feedback for the training of SPs and facilitators?

Our current research explores issues pertaining to the lived experience of giving feedback and challenges in practice that are not reported in the guidelines literature cited above.

## Methods

Qualitative methodology based on a constructivist grounded theory approach was adopted. This approach involved the researchers seeking to construct theory through engagement with and interpretation of the participants’ stories [21]. Constructive grounded theory offers a set of principles, not rules, where “neither data nor theories are discovered. Rather, we are part of the world we study and the data we collect. We construct our grounded theories through our past and present involvements and interactions with people, perspectives and research practices” [21].

### Sample

Following ethical approval from Queen Mary University of London, invitations to participate were sent via email and through advertisements with London-based simulated patient acting agency to ensure a purposive sample. The participants included simulated patients (actors) from these agencies with a minimum of 2 years’ experience working in variety of medical schools’ undergraduate communication skills programmes, primarily within London but also elsewhere. A total of 30 participants took part in this study. All but four had received formal training for this work. Their demographic characteristics are summarised in Table 2. The sample size was determined iteratively from the data, with data collection being deemed sufficient when categories could be identified and relationships between themes explained coherently. We determined we had achieved theoretical sufficiency or saturation of our sample after the six focus groups [22] when no new themes or codes were identified.

**Table 2:** Summary of demographic characteristics of the sample

	Total
**Gender**	
Male	14
Female	16
**Age Range**	
18–24	2
25–34	0
35–44	5
45–54	9
55–64	11
65–74	3
**Ethnicity**	
White	15
Mixed/Multiple	3
Asian/Asian British	4
Black/ African/ Caribbean/ Black British	4
Other	0
**Years of Experience**	
2–4	0
5–10	3
10–15	8
15–20	9
20 +	10

### Study design

Focus groups were utilised as they enabled a multiplicity of experiences and perspectives to be gathered within a group context. Thereby, facilitating insights into SPs’ shared understanding of how to provide feedback in communication skills teaching and the ways in which they are influenced by others in a group situation. It also supports the exploration of the degree of consensus among SPs on how feedback should be provided [23].

Six focus groups were conducted each with 5 to 6 individuals per group for a duration of 90–120 minutes. The first and second author facilitated the focus groups which were audio-recorded and transcribed verbatim. The focus group question guide was developed in consultation with clinical communication facilitators and a small group of SPs (see Additional file 1). Participants were asked to describe individual experiences and prompted to consider contrasting experiences within the group, rather than directly answer questions from the guide, in order to mitigate the effects of imposing preconceived theory on their narratives and to maximise breadth and depth of data collection.

### Data analysis

**Fig. 1:**
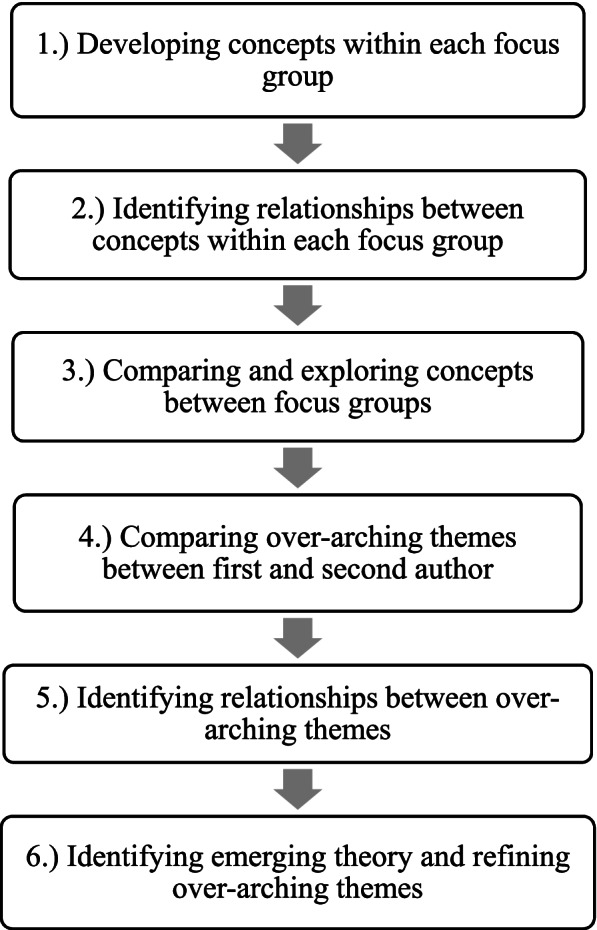
Overview of analytical steps

Data was analysed using a grounded theory approach, which involved a systematic set of techniques and processes that led to the identification of concepts and the building of theory from qualitative data [24]. It is primarily inductive in nature, whereby researchers move from the specific to the general to explain phenomena and encourages an openness to multiple explanations. The six focus groups were first analysed separately by RG (first author) and HW (second author) (both of whom are experienced academics in the field of communication skills teaching) to avoid bias in the interpretation and involved the following steps outlined in detailed below and summarised in Fig. 1.

1. Developing concepts within each focus group: Each focus group transcript was read and coded for concepts sequentially. Words, phrases or large blocks of data were abstracted under conceptual headings, for example, ‘lived patient experiences’.
2. Identifying relationships between concepts within each focus group: The relationships between concepts was then explored to begin to build theory. Concepts relating to feedback processes were categorised as a priori codes (meaning codes developed before analysing the data). The large majority of the coding framework was inductively derived.
3. Comparing and exploring concepts between focus groups: Data from different focus groups was then compared and contrasted through a process of ‘constant comparison’ as defined by Corbin and Strauss [24] to establish over-arching themes or categories. This constant comparison process of concepts with data from the different focus groups, in turn led to new concepts, for example the concept, ‘lived patient experiences’ became subsumed within an over-arching theme titled ‘cumulative experiences’, which included reference to ‘experiences of being a real patient’ of SPs when providing feedback during communication teaching.
4. Comparing over-arching themes between first and second author: RG (first author) and HW (second author) then compared and discussed over-arching themes to further refine interpretations, salient concepts and emerging theory, noting areas of similarity and differences. For example, the code ‘experiences of being a real patient’ which was formulated by the first author was defined as ‘bringing authenticity to the SP role’ by the second author.
5. Identifying relationships between over-arching themes: RG and HW then began to make tentative propositions about the relationships between emerging over-arching themes and how variation in the context might shape participants experiences. This is formally known as ‘axial coding’ as define by Corbin and Strauss [24]. RG and HW continually meet to review the axial coding (bringing of data together), discussing overarching themes and their relationships and considering their theoretical significance.
6. Identifying emerging theory and refining over-arching themes: As discussions matured, RG and HW made reflexive and theoretical memos (written records of the analysis). The process of ‘memo-ing’ guided theory building [25, 26]. This final phase also involved ‘selective coding’ meaning the identification and refinement of over-arching themes that incorporated other themes or superseded them in explanatory concepts. The relationships between these over-arching themes constitutes to substantive theory about the experiences of SPs which was conceptualised in an integrative diagram shown in Table 3.

**Table 3:** Cumulative experiences

**• Being a simulated patient** – experiential, emotional, interpersonal interaction. **• Having been a simulated patient** – the prior experience and expertise that is retained. **• Being a repeated simulated patient** in the same role in different communication teaching sessions that are happening concurrently. **• Being both a participant and an observer** – characterised as the ‘third eye’ where simulated patients’ continually mentally retain key communication aspects to recall in their feedback. **• Giving in-role feedback** – demonstration of raw emotion and an insight into the character of the patient. **• Giving out-of-role feedback** – providing constructive feedback from the patient’s perspective. **• Being a real patient** – utilising personal experiences when portraying characters. **• Helping students experience particular teachable moments** – understanding the learning objectives of the session, interpreting patient scripts and accurate prompting and probing. **• Assessing as a simulated patient** – the process of ensuring and maintaining standardisation.	

Data analysis was facilitated and supported by a combination of by hand analysis to retain the contextual nature of the data and using Nvivo 10 software [26].

## Results

Five over-arching themes were identified from the data analysis; 1) Feedback processes, 2) Challenges in providing feedback, 3) Cumulative experiences, 4) Web of interpersonal relationships and dynamics and 5) Portraying the character and patient representations.

### Feedback processes

The findings highlighted the complexity of the feedback process in how effective feedback was understood, the language used to convey it, the ways of giving feedback (e.g. ‘in-role’ or ‘out-of role), the order in which feedback is given in the teaching context and the cumulative experiences SPs utilise to develop their knowledge and vocabulary on how to provide feedback.

Participants regularly made reference to describing “how they felt” when defining effective feedback. They expressed the necessity of prioritising emotions and feelings in their feedback, stating,

“It’s always about how they made me feel as a human being … it’s all about me and it’s very personal.” (Focus group 3)

Whilst participants did refer to the importance of retaining specific details of the communication process such as the language used or non-verbal aspects, greater emphasis was placed on the interpersonal emotional connection between them and the students. Participants varied in their use of language to give feedback, either describing in first person, “I felt”, third person, “the character felt” or alternating between the two. Some participants described the benefit of using the third person to detach themselves from the character that was portrayed. Conversely, others argued the feedback was more personal and authentic when given using the first person.

The majority of participants generally expressed a strong preference for ‘out-of-role’ feedback, where they were able to return to being themselves, rather than giving feedback whilst portraying the character. They described that this better enabled the delivery of constructive feedback that was filtered to account for the students and the facilitator’s receptivity to the feedback. ‘In-role-feedback’ was considered as “raw unfiltered emotions and reactions” that characterised the feelings and temperament of the ‘patient’, with little regard for how they might affect others. Participants also reported the significance of using a lay person’s words in providing feedback, rather than technical communication jargon that was employed by the facilitator and the students, as this was more representative of the patient.

Participants frequently preferred the ‘sandwich style’ method for delivering feedback, comprising of first giving positive, followed by negative and then again positive feedback. Their insistence on this method stemmed from the notion that providing feedback as a SP can potentially be construed as an act which threatens students’ self-esteem, particularly in the context of communication teaching where students’ performance is observed by their peers. Feedback conversations and evaluative narratives were continually influenced by a persistent rhetoric among the SP community of “always being positive” in their feedback. This tendency to protect the self-esteem and self-efficacy of the student was often made at the expense of providing ‘fully honest’ feedback. By contrast, some participants began with their feedback by facilitating the students to self-assess their performance, reporting that “rather than you take on the mantle of deconstructing their performance, I think it is collaborative. ‘How was it for you? How was it for me?’” (Focus group 4).

After the role play is enacted, the facilitator conducts feedback with student self-appraisal, SP, observing students and facilitator. Participants described how this order of feedback varied depending on the facilitator’s preference, which in turn affected how feedback was given and received. Participants reported differing personal opinions on order of feedback, although many expressed a preference for going last to enable them to gauge the student’s level of insight and readiness to receive feedback and the facilitator’s preference for how feedback should be given. Participants described how they actively built upon their cumulative experiences of being an SP to develop their understanding and vocabulary to frame feedback.

### Challenges of providing feedback

The findings highlighted an array of variables influencing the delivery and content of SP feedback. Participants stressed a distinction between the SP and the student participating in the role play, with the students and facilitator observing the role play. The former pair characterised an ‘active’ interpersonal experience, whereas the students and facilitator observing the interaction were “spectators” to that experience.

“There is a difference between the observational aspect of watching the role play, which the facilitator is doing along with the rest of the students, and someone who is actually engaged in the emotional interaction of the role play. I think that is where we can give real benefit. We have a very different perspective and we will all have a very different perspective. They might cover all the correct ground, but even a tutor can’t tell you how you feel and you know exactly as an actor how you feel in that moment. I think there needs to be more of an understanding that there are two slightly different experiences from spectating and being involved directly in the conversation.” (Focus group 4)

These distinct standpoints gave different vantage perspectives on the communication process and assessment of the student’s performance, which often resulted in contradictions, as illustrated below.

“Only we as the role player know how it feels. I have also been in a situation where the facilitator has said, ‘I thought that there was terrific empathy there,’ and I have thought, no. I really didn’t feel that because it was me. As an observer that facilitator thought that there was, but I didn’t feel it at all.” (Focus group 4)

Dissonance sometimes arose between the facilitator and the SP. Many participants felt a prevailing expectation from facilitators that SPs were to provide largely positive feedback, even if it contradicted their assessment of the interaction. They attributed this to the facilitators’ anxiety in having to protect the students from experiencing adverse emotions and reactions to negative feedback. They also referred to a culture in medical education of constant summative assessments with negative feedback viewed as failing. One participant described their experience below:

“The tutor sort of intervened. I thought I was being positive, but intervened to get me to say something which was just flat-out, ‘but it was good,’ or something … I think the tutor wanted everything prefaced with, ‘it was great,’ and then go into it, and it wasn’t, that particular one. That was my disagreement with the tutor. It felt like I was having to dilute it or manufacture generic positive feedback for the sake of self-esteem or protection.” (Focus group 2)

Some participants conveyed that these contradictions were sometimes exhibited by facilitators through implicitly disregarding the validity of the SP’s feedback, for example:

“What I dislike and what is not effective is when I am giving a feedback and then it is disagreed by the facilitator. That has happened before within this room and that doesn’t set us up. ‘He is only an actor. What does he know about?” (Focus group 6)

A recurring theme was the challenge in providing feedback to those students struggling with their communication skills. The careful balancing act of providing the right level of positive and negative feedback was continually reported and appeared to be modulated by a host of factors. These ranged from the student’s level of insight, their receptiveness to receiving negative feedback, the openness of their peers to giving honest feedback and the facilitator’s preference for how feedback should be given. Being ‘fully honest’ when providing feedback was a debated practice among SPs, with some reporting an absolute need for honesty to foster improvement. Others were hindered by what they perceived as the institutional culture of medicine in not wanting to acknowledge and experience failure and the hierarchical educational culture which impeded bidirectional feedback between the SP and the student.

“They are quite fragile’ the tutors say. ‘Yes, but they have to learn’. You are not going to go in there and destroy them, sometimes these are really personal things. If you are told anything to do with, ‘I didn’t connect with you,’ it hurts a bit. but if you can somehow pull up a point of something that they could do to improve … ‘No, that is up to me. We just want positive stuff’ is the response I sometimes I get from the tutor.” (Focus group 3)

The artificial nature of a simulated environment and the vulnerability of the students in being observed by their peers was thought to create a barrier to full engagement in the process. Participants repeatedly noted that if the consultation had not gone well, students would be quick to blame the inauthenticity of the situation and begin to question whether ‘real patients’ would behave as depicted by the SP:

“They say that it is not real. The inauthenticity of the situation is often used as a defence. ‘In reality it wouldn’t happen like this so then I wouldn’t do that.’ They will then say ‘this is unrealistic, this would never happen.’ What I find really difficult about giving feedback? Resistance. If they are super resistant to what I am saying, they are almost cynical about the process? ‘This is not real...it wouldn’t really be like that.’” (Focus group 3)

### Cumulative experiences

The findings illustrated the multifaceted role of a SP when involved in communication skills teaching. These different experiences were cumulative, concurrent and were susceptible to a range of contextual and socio-cultural influences. The findings highlighted a set of core experiences outlined in Table 3.

Participants frequently noted the distinctive experiences of the SP in their roles and the differing perspectives they bring compared to those of the facilitator and students, which presents both inherent benefits but also considerable challenges. As described by one participant.

“There is a difference between the observational aspect of watching the role play, which the facilitator is doing along with the rest of the students, and someone who is actually engaged in the emotional interaction of the role play. I think that is where we can give real benefit. We have a very different perspective and we will all have a very different perspective. They might cover all the correct ground, but even a tutor can’t tell you how you feel and you know exactly as an actor how you feel in that moment. I think there needs to be more of an understanding that there are two slightly different experiences from spectating and being involved directly in the conversation.” (Focus group 4)

Over the years SPs had been working in a variety of programmes, in the same or different roles and in formative or summative contexts. They described how they evolved expertise and insights from previous interactions, sometimes repeating the same role in different sessions, honing their ‘third eye’ whereby they mentally retain points to recall in feedback, giving ‘in-role’ and ‘out-of role’ feedback, sometimes drawing upon their own experiences as a patient, interpreting the patient scripts, linking their prompts and cues to the learning objectives for ‘teachable moments, and standardising their delivery and assessment.

These different experiences were cumulative, concurrent and susceptible to a range of contextual and socio-cultural influences. They highlight the many facets of fulfilling the role of an SP in communication skills learning.

All SPs in this study had more than 5 years’ experience of medical role-play (Table 2). This can inform their knowledge about clinical features of patient scenarios and the nuances of teaching communication skills, whilst building their vocabulary for feedback delivery. They described the duality of their role in being both a participant and an observer during the simulation, and its importance in providing effective feedback.

Furthermore, participants described utilising their real-life experiences as patients in playing roles when these roles resonated personally. As one participant remarks “as simulated patients sometimes we have the disease that we’re pretending to have, sometimes we’ve actually been through it.” Drawing upon real life experiences was deemed essential in fostering effective feedback.

### Web of interpersonal relationships

Participants described how their experiences and provision of feedback was modulated and influenced by a web of relationships; namely the relationship between the SP and facilitator, the SP and the students and the facilitator and the students. The relationship between SP and facilitator was deemed most influential to the quality of the other two relationships. Participants described this relationship variously. They often referred to the hierarchical difference and the importance of “tip-toeing” or “not stepping on each-other” roles.

“I think as an overall learning experience it is like as an actor in a production. You can’t really be better than the director. You can be very slightly better than the director because you are always attuned to the play. You can’t be better than the facilitator because if you are, you undermine the facilitator.” (Focus group 1)

The relationships between the SP and the facilitator that worked the best was described as a “negotiated, equal partnership”. This involved an active acknowledgment of the value SPs bring, discussing feedback processes and clarifying information in the patient script in a briefing beforehand, and establishing mutual respect. One participant expressed it as:

“You are making a journey together and you are collaborating. Basically what we do is we are codifying things that most people take for granted in everyday life, then we build up a bank of vocabulary and ways to describe these very natural interactions.” (Focus group 5).

The relationship between the facilitator and the students was described as the pre-requisite that influenced the quality of SPs’ relationship with the students in creating a safe learning environment in which to practice. Participants argued that facilitators were key in adequately preparing students for participation, creating an atmosphere of safety where the patient’s perspective is valued, and encouraging students to try, be allowed to make mistakes and repeat practice.

This web of interpersonal relationships, in particular the relationship between the SP and the facilitator, was also influenced by the unique context of communication skills teaching. Typically, communication teaching in medical schools involves a number of small group sessions running simultaneously and SPs may be required to rotate between the groups. Whilst SPs play the same role, the learning environments they enter in quick succession differ, affecting how the role plays out and resulting in different feedback. One participant describes.

“It is very difficult if you go into a room and you get used to … , going from room to room to room, you cannot help in your head almost compare the preparation that has been done to facilitate the group before you get there and then sometimes it is quite difficult because you go into a room and the tutor is running it differently, asking for different types of feedback or the student may be nervous.” (Focus group 2)

These rotations and the unique context of teaching communication skills necessitated adaptability from SPs in having to adjust to different facilitator styles, feedback preferences and the dynamics of student groups. Participants emphasised the significance in not under-estimating the importance of contextual factors in their influence on the quality of these different relationships. These hidden social rules and implicit expectations which are different to each tutor require SPs to have the ability to identify and navigate these subtle dynamics within a short space of time. From the participants accounts, it appears they need to rapidly adapt to a different set of rules and expectations when they move across the hallway to a different tutor, which can create considerable potential for misunderstanding and uncertainty.

### Portraying the character and patient representations

Participants talked of their experiences in portrayal of the patient character. They commonly shared and exchanged perspectives on interpreting patient scripts with each other. Whilst clinical facts were a given, the personal aspects lacked detail and SPs improvised the patient’s characteristics such as their personality, demeanour, temperament and social situation. The findings also suggested many patient scripts were devoid of information on the learning objectives of the teaching session.

“Sometimes when you get the brief it’s written in medicalised way … most patients sometimes blurt everything out. We don’t know that knee infection is connected to a sexually transmitted disease. Sometimes character briefs are quite good because they tell you roughly how you might react in different situations, but we have to do a lot of filling in the gaps.” (Focus group 2)

Participants were generally assigned roles based on their personal characteristics such as gender, age, race and weight. This often resulted in identification between the character being portrayed and the SP, making it simpler to draw on real-life experiences. The subtleties in how SPs’ personal characteristics affected the interaction and the feedback provided was raised by participants with some reporting that they would become more sensitive, attuned and reactive. Below is an account from a participant playing roles associated with motivational interviewing.

“I am considerably overweight and often play the lifestyle/motivational interviewing roles. How people handle talking about being overweight must go to me at some level because I am overweight. I am assuming there must be some reaction to that. I don’t think anybody has ever dealt with it insensitively, but if they did I would probably notice. That is just because in that particular situation there is a parity between me and the patient.” (Focus group 4)

Participants raised the issue that the way certain characters were asked to be portrayed inadvertently stereotyped patients, “Asian and type II diabetes, Black patients usually seen as aggressive.” Some participants reported that facilitators would describe SPs as “difficult patients”, giving a negative representation of patients. One participant recalls:

“‘This is a difficult patient.’ You think, I am representing a patient that is very real. They are not thinking they are difficult. They might just be ill. They might be crying out for something. That should come from the facilitator as well.” (Focus group 5)

These negative remarks made participants approach their feedback with apprehension as they wanted to avoid contributing to preconceived ideas and biases about particular patients. Participants noted that they “symbolise the outside world” of patients and disparaging comments were harmful in shaping students’ perceptions of patients. Rather than depicting a patient as “difficult”, they thought students could be encouraged to acknowledge that “this is a patient with difficulties.”

**Fig. 2:**
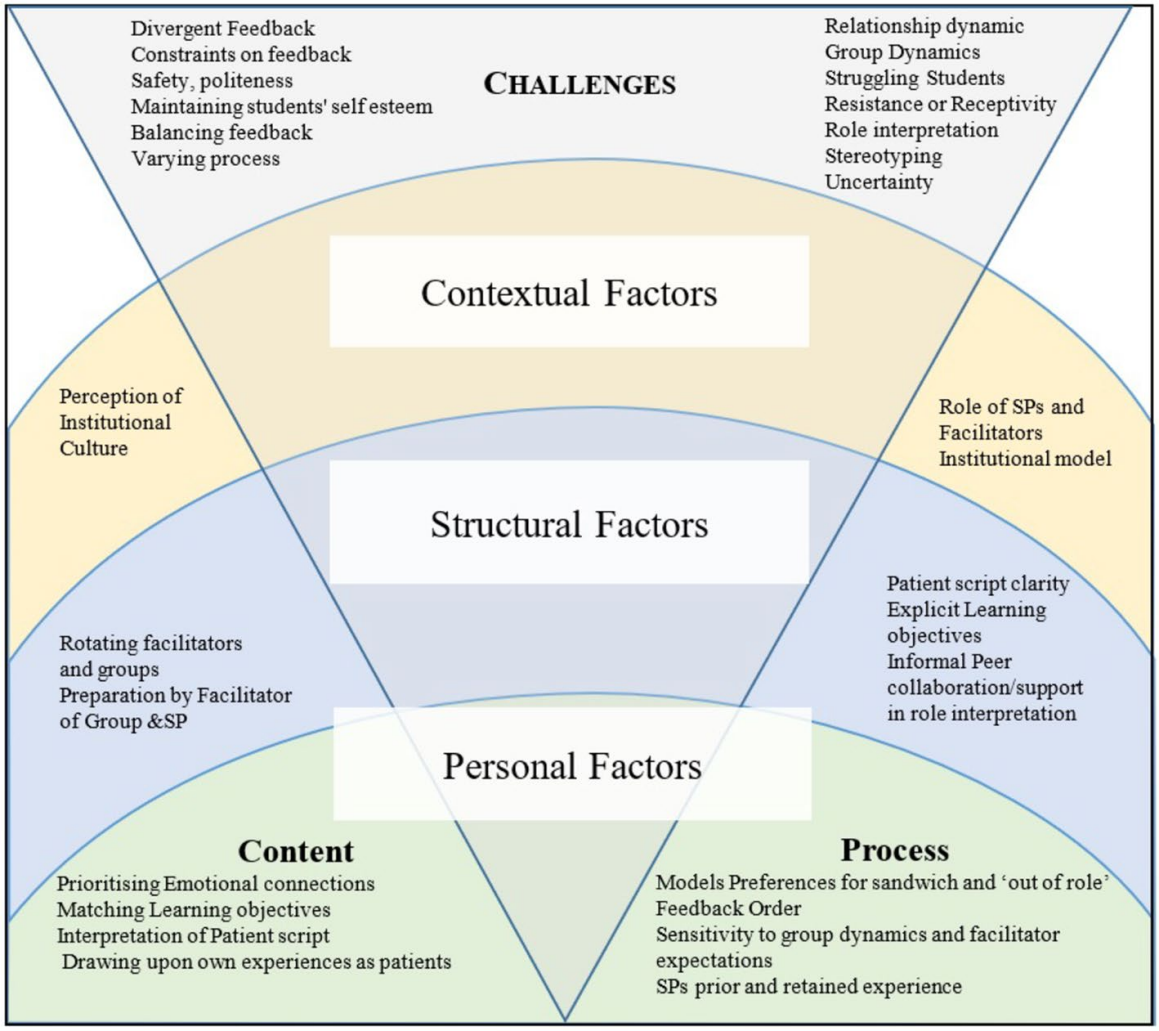
The socio-cultural landscape of SP feedback experiences and views

The socio-cultural influences on SPs feedback derived from the findings are summarised in Fig. 2 which draws together the interplay of personal, structural and perceptions of institutional factors to illustrate a landscape of feedback moderators.

## Discussion

Within the literature on feedback, there is an absence of research from the perspective of SPs. Their experience of providing feedback is a complex process and influenced by an assortment of contextual and socio-cultural factors highlighted in this study.

Our findings suggest that whatever model of feedback may be adopted by institutions, simulated patients might have their preferences for how and what to provide feedback on. They predominantly, in this sample, felt their role was to provide feedback on the emotional connection within a consultation. They also revealed challenges pertaining to the relationship with different facilitators, the group dynamics and student receptivity and the adjustments these factors required of them. Not only do SPs have to play a role authentically and accurately, they have to remember the interaction points that help or hinder the consultation and be sensitive to the social context in which they are providing feedback.

Communication skills programmes with SPs have been running for many years with institutions using a variety of preferred models of feedback and bespoke training programmes for SPs and faculty. The findings from this study may not be typical of SPs in other settings. Nevertheless, the significance of the relationship between facilitator and SP and the influence of group dynamics are issues that are broadly applicable to any learning environment.

Our study suggests SPs regard their primary role as providing a transparent description of one’s feelings and the perceived emotional connection in a clinical encounter. In contrast to traditional notions of feedback which typically focus on describing non-emotional aspects of students/trainees performance, SP feedback appeared to be permeated by the emotions experienced [27]. The experience of providing feedback on interpersonal connection is generally unreported, with the majority of papers focusing rather on how to manage the emotional responses to receiving feedback [28].

It is well established that the student–tutor relationship plays a key role in students’ learning [29]. Consistent with this, our findings suggest SP feedback is also influenced by relationships, students’ receptiveness to receive feedback, perceptions of credibility and the institutional culture [18–20]. This study is unique in illuminating the complex and dynamic relationship between SPs, facilitators and students that was frequently highlighted and perceived to impact on the quality of the educational experience. Facilitation models and session structures vary across institutions and SPs may have to identify and navigate these subtle dynamics within a short space of time, rapidly adapting when they switch to a different setting, facilitator and group. This challenge requires a degree of flexibility and judgement which may include holding back in order not to undermine and risk safety in the group, but in so doing might threaten learning opportunities. A peer community appears to exist whereby SPs share ideas to refine, clarify and interpret roles and group process.

The sandwich style of feedback appeared to be a preferred method and resonated with Pendleton’s approach [30]. It also aligned closely with the concept of ‘coaching’ whereby coaches work with trainees to improve their performance by guiding them to the next level. Providing feedback was considered a potentially threatening act which is consistent with politeness theory, which assumes that conversations are potentially ‘face-threatening’ acts to the hearer or speaker [31]. ‘Face’ is categorised as positive (the need to project a positive image) and negative (freedom to act without imposition). SPs experiences with facilitators suggested a perceived expectation to promote and predominately use positive feedback. This is linked to politeness concepts where prioritisation of self-image and self-esteem might impede honest constructive narratives and feedback [32, 33]. The belief amongst some SPs that only positive feedback was expected was very concerning. This was described to be a potential constraint and resulted in SPs approaching feedback conversations particularly on areas for improvement with trepidation. Aligned to this was the finding that SPs felt their feedback sometimes contrasted with facilitators’ observations and rather than providing a fruitful area for discussion was disregarded or devalued. How to deal with conflicting feedback is an area that both SPs and facilitators could usefully explore in training. Models that focus on constructive feedback for achieving goals and learning objectives rather than positive and negative concepts [34, 35] and how to frame this linguistically are helpful in this respect. The disproportionate emphasis on summative assessments in medical education may also inadvertently instil binary perceptions of students’ performance as either positive or negative, rather than on a spectrum of continual progression and growth [34]. Re-focusing the learner beyond binary outcomes of pass and fail should be used to in feedback to inform future learning and communication with patients. Our findings indicate a need to revisit feedback training of SPs and facilitators that includes discussion of the respective roles of facilitator and SP and how to work collaboratively around different perspectives.

Whilst the designer of the educational programme will decide on in-role or out of role feedback, SPs in this study generally preferred ‘out-of-role’ feedback. It provided distance from the emotions of the patient and enabled them to provide more constructive feedback. However, this stance can reduce the impact of the patient’s ‘in the moment’ responses which can be very useful. One approach used to balance the tension between providing a patient perspective without the raw emotion is to utilise a ‘role neutral’ position [35]. The SP remains in role but does not stay in the emotional state experienced in the role play, ensuring the emotion does not interfere with learning. The comment by some SPs that bringing their own real-life experiences to bear in feedback was essential may worry faculty concerned about bias but illustrates that role players believe their authentic experiences are valuable.

The model developed in this study maps out the potential interplay of personal, structural and institutional factors from the SP perspective to illustrate a landscape of feedback issues. This study provides valuable new insights including the pivotal interaction between the SP, facilitator and learner.

### Limitations of the study

The SPs involved in this study work across various healthcare institutions, predominantly medical schools predominantly within London and experiences of SPs in other medical schools may be different. The majority were between 45 and 64 with over 15 years’ experience of medical role-play and younger SPs may have different perspectives.

## Conclusion

Despite the evidence to show the value of SP feedback [36], this is an under-researched area. This study has revealed that SPs’ feedback to medical students is a complex and nuanced process influenced by relational and structural factors. The findings have important implications for discussions about how to maximise the value and quality of feedback provided by SPs. Complementary research we have undertaken into facilitator perceptions of SP feedback will be the subject of a forthcoming publication and together will provide useful ideas for faculty development and SP training. This study raises questions about the roles of SPs and facilitators and their working relationship, so far unreported in the literature. It provides evidence that, in addition to feedback training, opportunities to review collaborative working with facilitators is desirable to address some of the challenges identified.

## Supplementary Information

**Additional file 1.** Example of Focus Group Guide for Simulated Patients (SPs).

## Data Availability

The datasets used and/or analysed during the current study available from the corresponding author on reasonable request.
