# Supplementary material for: Experiences of simulated patients in providing feedback in communication skills teaching for undergraduate medical students
Source: BMC Med Educ. 2022 May 3;22:339. doi: 10.1186/s12909-022-03415-6 (PMC9066858; doi:10.1186/s12909-022-03415-6)
Supplement: Supplementary file 1 — Additional file 1. Example of Focus Group Guide for Simulated Patients (SPs). [file 12909_2022_3415_MOESM1_ESM.docx]

**Appendix 1: Example of Focus Group Guide for Simulated Patients (SPs)**

| **Time (mins)** | **Research questions** | **Follow-up/ prompt questions** |
| --- | --- | --- |
| 0 -5 | Introduction | Reiteration of information on the participant sheet  Explanation of focus group and ground rules |
| 5 - 25 | What’s effective feedback for you? | - What key aspects influence your delivery of effective feedback? - What do you find challenging about giving feedback to medical students? - Do the learning objectives of the teaching session play a role in your feedback? - What contexts (i.e. history taking, assessment - OSCEs) are you involved in teaching? - What are your experiences of being trained to give effective feedback? |
| 25 - 50 | What are your experiences of in-role and out-of feedback? | - What are the differences (between in-role and out-of role) feedback for them and for the learner? - Do you provide feedback in the first person or from the patient’s perspective? (i.e. I felt versus patient X felt…) - Does the role you play affect the feedback given to medical students? - Do you feel a difference in your feedback when playing a new role compared to a role you have played several times? |
| 50 - 75 | How is your feedback as a simulated patient different to the tutor? | - How is the language used in your feedback (as a simulated patient) different to the tutor? - How does your feedback (as a stimulated patient) compared to the tutor’s feedback affect medical students’ learning? - Have you experienced a situation when your feedback is in conflict with or significantly different from the tutor’s feedback? How did you feel? How was this managed? |
| 75 - 80 | End – Reiteration of confidentiality and further information on the study | |
